# Outlier Populations: Individual and Social Network Correlates of Solvent-Using Injection Drug Users

**DOI:** 10.1371/journal.pone.0088623

**Published:** 2014-02-11

**Authors:** Souradet Y. Shaw, Ann M. Jolly, John L. Wylie

**Affiliations:** 1 Department of Community Health Sciences, University of Manitoba, Winnipeg, Manitoba, Canada; 2 Public Health Agency of Canada, Ottawa, Ontario, Canada; 3 Dalla Lana School of Public Health, University of Toronto, Toronto, Ontario, Canada; 4 Cadham Provincial Laboratory, Winnipeg, Manitoba, Canada; 5 Department of Medical Microbiology, University of Manitoba, Winnipeg, Manitoba, Canada; UCL Institute of Child Health, University College London, United Kingdom

## Abstract

**Objective:**

We previously identified a high prevalence of Hepatitis C (HCV) amongst solvent-using injection drug users (S-IDU) relative to other injection drug users within the same locality. Here we incorporated social network variables to better characterize some of the behavioural characteristics that may be putting this specific subgroup of IDU at elevated disease risk.

**Methods:**

A cross-sectional survey of at-risk populations was carried out in Winnipeg, Canada in 2009. Individuals reporting any history of injection drug and/or solvent use were included in the study. Associations between subgroup membership, infection with HCV and HIV and individual and social network variables were examined.

**Results:**

In relation to other IDU, S-IDU were more likely to be infected with HCV, to report ever having shared a syringe, and to associate with other IDU. They were further differentiated in terms of their self-reported sexual orientation, ethnicity and in the injection drugs typically used.

**Conclusion:**

Solvent use stands as a proxy measure of numerous other characteristics that put this group of IDU at higher risk of infection. Provision of adequate services to ostracized subpopulations may result in wider population-level benefits.

## Introduction

Compared to HIV, there have been relatively fewer studies focusing on the public health impact of Hepatitis C (HCV)[1]. This is despite the substantial burden HCV infections pose to health systems. A US study published in 2011 estimated over 5 million individuals as having chronic HCV infection; in comparison, in 2009 the Centers for Disease Control estimated 1.2 million people living with HIV[2], [3]. These two data sources place the death toll at similar levels, with 11,000 deaths attributed to HIV vs. 8,000–11,000 deaths for HCV. The situation in Canada is not unlike that of the US; one recent modeling study compared the burden of HCV and HIV using premature mortality and disability-adjusted life years[4]. This study estimated HCV contributed 8,823 years of premature mortality in the province of Ontario, as measured by years of life lost (YLL), compared to 5,036 YLL for HIV[4].

Although well-understood differences exist in their actual transmission, both pathogens demonstrate similarities in the populations they typically affect. These populations, such as injection drug users (IDU), tend to be thought of and characterized as marginalized, relative to mainstream society[5]–[7]_ENREF_5. Over half of prevalent HCV infections in Canada, and up to 75% of incident HCV infections are due to injection drug use[8]. Some of this elevated risk is linked to proximate factors in which parenteral exposure to blood-borne pathogens occurs via contaminated syringes and injection equipment. However, it is well-known that certain subgroups or “outliers” exist within already marginalized populations. A review of HCV found rates were highest amongst Canadian and Australian Aboriginal IDUs compared to non-Aboriginal IDU[9]. Findings of this type suggest the influence of more distal micro- and macro-level factors which significantly elevate infection risk within specific subgroups. In the case of ethnicity, these more distal factors could involve aspects of stigma, discrimination and/or decreased access to health care services[9].

A significant amount of resources have been mobilized to prevent sexually transmitted and blood-borne infection (STBBI) transmission, meeting with varying degrees of success[10]–[12]. For example, although syringe exchange programs (SEPs) have been considered effective in curtailing widespread epidemics of HIV/HCV among IDU, the effectiveness of SEPs in curbing syringe-sharing *per se* has been heterogeneous across IDU populations[11], [13]–[20]_ENREF_80. Socio-epidemiologic explanations for this moderation of SEP impact acknowledge the influence of more distal contextual factors, such as relationships between sexual partners and friends[21]–[27]. Thus, just as transmission risk differs between subpopulations, the effectiveness of interventions would show the same variability, such that a “one-size-fits-all” approach would be intractable with respect to the planning of STBBI interventions[17], [27]–[32].

In our locality of Winnipeg, Canada, and despite relatively low HCV rates among IDU (in comparison to IDU from other Canadian cities)[33], we have previously demonstrated that HCV prevalence was 81% among Aboriginal solvent-using IDU (S-IDU), or threefold the odds, compared to non-solvent using Aboriginal IDU[34]. We further showed that recent syringe-sharing was 10 times higher among S-IDU[34]. Although behavioural patterns such as this can be taken as an immediate potential cause for elevated HCV rates amongst S-IDU, the underlying reasons for why syringe-sharing is higher remain unknown. However, given the confluence of historical oppression, and socio-economic inequities which mark chronic solvent-use in Canada[35], [36], the extreme social marginalization and subsequent isolation of S-IDU is likely an important contributor[37], [38]. The social milieu in which S-IDU find themselves may also be more homogeneous, at least within the context of comprising similarly marginalized individuals[37]. This combination of marginalization and isolation may lead to social mores which favour riskier group behaviours, and may then ultimately lead to higher pathogen prevalence[17], [27], [39]. Insights into the composition of S-IDU networks can help inform prevention and intervention efforts of marginalized groups other than S-IDU, as similar factors are thought to underlie formation of subpopulations who are systematically underserved by public health[40], [41].

In the present cross-sectional study that took place in Winnipeg, Canada, we have expanded on our earlier work by extending analysis of solvent use and injection drug use to both Aboriginal and non-Aboriginal users, and to also incorporate participants' social network characteristics. The latter was intended as an exploration of the social milieu of S-IDU to better understand potential distal factors influencing the level of syringe-sharing amongst S-IDU, or otherwise putting S-IDU at elevated risk for HCV. We hypothesized that just as individual-level factors, such as syringe-sharing, differed between S-IDU and IDU, differences would also be seen amongst the egocentric risk network members (i.e., the people with whom the respondent had regular contact) with whom S-IDU and IDU groups typically interact.

## Methods

Data for this analysis were extracted from a 2009 cross-sectional survey (SNS III) conducted in Winnipeg, Manitoba, Canada (pop. 675,000). The overall study was intended to measure social interaction patterns between members of populations considered at higher risk for STBBIs. Recruitment was via respondent driven sampling with recruiters instructed to provide recruitment coupons to members of their social network who they perceived as being at risk for STBBIs. Recruitment took place over an 11-month period from January to December 2009, with all interviews and specimen collection being carried out by one research nurse. This nurse had also identified potential interview sites (e.g. within local clinics or resource centres) prior to study implementation. RDS coupon distribution was voluntary and no secondary incentives were provided for enrollment of others into the study. Three coupons were provided to each study participant for purposes of recruitment. To initiate recruitment, the research nurse selected 22 individuals as RDS seeds. Using specific risk groups as examples, 15 of these individuals were IDU; 4 were street-involved youth, 9 were sex workers, and 4 were men who have sex with men, with the total exceeding 22 as some individuals were members of more than one of these groups. As noted above, the SNS III survey was meant to better understand interaction patterns between many different types of groups at risk for STBBI. Thus, study participants were instructed to recruit other friends or family members who they believed practiced some of the risk behaviours they had been questioned about during their interview.

Since STBBI status was unknown to the interviewer at the time of recruitment, our sample included individuals who were both aware and unaware of their STBBI status. A lower age limit of 14 was used for recruitment; however, after exclusions (see: Study Exclusions and Outcome Variable), our sample only included those 18 years and over. Potential participants made telephone contact with the study nurse, who administered all surveys in-person, at a location of their choosing. An honorarium was provided to study participants providing written consent. Participants either read the consent form themselves, or if they preferred, had the consent read to them by the study nurse. The study nurse made herself available for questions or discussion. Participants then were asked to provide signed consent if they understood the goals of the study, and agreed to participate. Participants were given the option of opting out of any parts of the questionnaire they wanted to, as well any of the biological tests performed. The questionnaire was divided into two sections. Section 1 consisted of questions based on the respondent's own characteristics, while section 2 elicited information on the respondent's egocentric network. Individuals listed a maximum of 10 people with whom they had had more than casual contact over the last 3 months. Prompts included friends, relatives and people with whom they had used drugs, had sex, resided or hung out with; variables from section 2 are referred to as egocentric network variables. Only those participants providing written consent were included in the study. The study design and consent process was approved by the Health Research Ethics Board of the University of Manitoba and the Winnipeg Regional Health Authority Research Review Committee. As Aboriginal persons were included in our study, and as biological specimens were collected, the Principal Investigator (JLW) of the study presented to the Assembly of Manitoba Chiefs Health Information and Research Governance Committee (HIRGC) in order to ensure that the research was respectful of OCAP (Ownership, Control, Access and Partnership) principles.

### Measures

#### Study Exclusions and Outcome Variable

Respondents were grouped into two categories: lifetime IDU only and both lifetime IDU and solvent use (i.e., S-IDU). Respondents were considered IDU if they answered positively to the question “have you ever injected any non-prescribed drugs”. Respondents were considered solvent-users if they answered positively to the question “have you ever sniffed any solvents”. From an initial sample of 600 respondents, the study sample was subsetted to only those individuals who were IDU, solvent users, or both (N = 378). Of these, 323 respondents gave consent to have their blood drawn; from these 323 respondents, only those who were IDU or S-IDU were included in the final analyses (N = 254).

#### HCV and HIV infection

HCV status was assessed using venous blood samples, tested at Cadham Provincial Laboratory (Winnipeg, MB). Specimens were tested for HCV with AxSYM Anti-HCV (Abbott, Mississauga, ON); HIV specimens were tested in the same manner, with the exception of the use of Advia Centaur HIV1/O/2 anti-HIV (Bayer, Mississauga, ON).

#### Socio-Demographic

Socio-demographic variables included: age; education, coded as ‘graduated or in school’, ‘dropped out in or before grade 9’ (typically 14 or 15 years of age in Canada), and ‘dropped out after grade 9’; and source of income, grouped as ‘regular source’, ‘welfare or other social assistance’ and ‘family/friends/other’. Binary variables were created for sex (female vs. male); gay, lesbian, bisexual, transsexual or transgendered (GLBTT) identity; and Aboriginal status, whereby those that identified as ‘First Nations’ or ‘Metis’ were categorized as Aboriginal.

#### Network Variables

For each member of their egocentric network, respondents were asked the following: whether this person was an active IDU (someone whom respondents knew had injected drugs in the last 6 months), if the respondent had drank alcohol with the network member, whether some other type of non-injection drug had been used, if the member had given/obtained drugs for the respondent, and whether resources (e.g., money) had been pooled with the member to obtain drugs. For the purposes of these analyses, results for each respondent were aggregated and binary measures were created to capture presence/absence of each network characteristic. Thus for example, the variable “Has an active IDU in network” indicates the presence of at least one IDU whom the respondent has known to have injected drugs in the last 6 months, and does not specifically refer to any single individual.

#### Drug-Using Characteristics

Where applicable (i.e., for respondents reporting injecting drug use), respondents were asked whether they had ever used syringes after someone else had injected drugs with that syringe. Although the questionnaire elicited information on a larger set of injection drugs, the following injection drugs were included in the current analyses, based on highest frequency: cocaine, Talwin & Ritalin (T&R), crack cocaine, morphine, crystal methamphetamine and heroin.

### Statistical Methods

Bivariate analyses were first used to characterize the socio-demographic and infection status characteristics of the S-IDU and IDU groups using χ^2^ tests of association. Next, unadjusted and adjusted multivariable logistic regression models comparing S-IDU and IDU were produced using an explanatory model-building approach. In this approach, all models were *a priori* adjusted for age, sex, and Aboriginal status. A three-stage model-building strategy was used: in the first stage, education, income source, GLBTT status, lifetime syringe-sharing, types of drugs injected, infection status variables and the network composition variables were each separately entered to assess associations with group membership (adjusted for *a priori* variables). Lifetime syringe sharing (as opposed to a more recent measure like 6 months) was used as more than half (54%) of IDU did not report any drug injections in the last 6 months. With the exception of infection status, variables were retained if they were significantly associated with group membership at the p<.05 level (see exception re: infection status variables, below). In the second stage, variables that met the above criteria were entered simultaneously (and adjusted for *a priori* variables). In the third stage, remaining variables which were not retained in stages 1 and 2 were re-entered into the model; re-entered variables were retained if they now met the criteria set out in the first stage of model-building. Generalized estimating equations (GEE) were used to correct for clustering within RDS chains, with an exchangeable correlation structure specified. Stata 11 was used for all analyses (College Station, TX).

In the model building process above, special considerations were made in the manner in which the infection status variables were handled. These variables were included in the bivariate analysis and at the first stage of the model-building process to demonstrate and confirm that the infection/S-IDU association we identified in our 2003 study population also existed in our 2009 study population[34]. In the multivariable analyses, given that the main intent of the present study was to identify demographic and behavioural differences between S-IDU and IDU (i.e. factors that could contribute to infection), infection status was specifically excluded.

## Results

A total of 254 individuals were included in the study, with HCV and HIV prevalence at 52% and 15%, respectively. In comparison, the prevalence of HCV and HIV among study respondents who did not report either solvent use or injection drug use (i.e., those respondents excluded from the present study, N = 222) was 7% and 2%, respectively. Comparing the 254 respondents who were included in the study to the 55 respondents who did not consent to blood tests revealed no significant differences by Aboriginal ethnicity (p = .351), sex (p = .058), source of income (p = .189) and LGBT status (p = .333). Those who did not consent were younger (p = .043), and were more likely to have reported only injection drug use in their lifetime (57%, p = .004).

Of those respondents included in the study, 65% (n = 164) were S-IDU and 35% (n = 90) had only used injection drugs in their lifetime (Table 1). From Table 1, it can be seen that group membership differed significantly by Aboriginal status (p<.001), HCV (p<.001) prevalence, and the presence of active IDU in the network (p<.001). HCV was highest among S-IDU (60%); S-IDU were most likely to report an active IDU in their network (49%).

**Table 1:** Socio-demographic characteristics of lifetime solvent and injection drug user (IDU) groups (N = 254).

	IDU Only (n = 90)	Solvent and IDU (n = 164)	*P*
	No. (%)	No. (%)	
Education (N = 249)			
Graduated/in school	28 (31.8)	40 (24.8)	.*187*
Dropped out < = Gr.9	27 (30.7)	68 (42.2)	
Dropped out> = Gr.10	33 (37.5)	53 (32.9)	
Income (N = 254)			
Regular	19 (21.1)	22 (13.4)	.*209*
Welfare, etc	57 (63.3)	120 (73.2)	
Other/Family/Friends	14 (15.6)	22 (13.4)	
			
Female (N = 248)	33 (37.1)	74 (46.5)	.*149*
			
GLBTT (N = 254)	15 (16.7)	32 (19.5)	.*576*
			
Age (N = 253)			
<25	19 (21.1)	23 (14.1)	.*402*
25–29	10 (11.1)	16 (9.8)	
30–39	21 (23.3)	50 (30.7)	
40+	40 (44.4)	74 (45.4)	
			
Aboriginal (N = 254)	52 (57.8)	134 (81.7)	*<.001*
			
HCV (N = 254)	35 (38.9)	98 (59.8)	*<.001*
			
HIV (N = 254)	14 (15.6)	23 (14.0)	.*741*
			
Has IDU who shot up in last 6 months in network (N = 248)	21 (24.1)	78 (48.5)	*<.001*
			
Has drank alcohol with someone in network (N = 248)	60 (69.0)	108 (67.1)	.*762*
			
Has used some other type of non-injection drug with someone in network (N = 248)	56 (64.4)	110 (68.3)	.*527*
			
Has someone who has given/obtained drugs in network (N = 248)	46 (52.9)	90 (55.9)	.*648*
			
Has pooled resources with someone in network (N = 248)	44 (50.6)	90 (55.9)	.*422*
			
Injection drugs (N = 254)			
Cocaine	71 (78.9)	131 (80.0)	.*852*
Talwin & Ritalin	34 (37.8)	106 (64.6)	*<.001*
Crack cocaine	27 (30.0)	38 (23.2)	.*233*
Morphine	45 (50.0)	57 (34.8)	.*018*
Crystal methamphetamine	17 (18.9)	22 (13.4)	.*247*
Heroin	23 (25.6)	30 (18.3)	.*173*
			
Shared syringes after injection (ever) (N = 243)	31 (36.5)	85 (53.8)	.*010*
			
Solvent types (ever) (N = 164)			
Lacquer	–	78 (47.6)	
Paint thinner	–	31 (18.9)	
Nail polish	–	43 (26.2)	
Gasoline	–	61 (37.2)	
Network size (mean [median, (IQR)])	5.5 [5 (3-7)]	5.9 [6 (4–7)]	.*258*

IDU: Injection drug users; GLBTT: Gay, lesbian, bisexual, transgendered, and two-spirited; IQR: inter-quartile range.

Comparing S-IDU against IDU-only, S-IDU were more likely to report ever using T&R (65% vs. 38%, p<.001) and less likely to report morphine use (35% vs. 50%, p = .018). Finally, S-IDU were more likely to report lifetime sharing of syringes after injecting drugs (54% vs. 37%, p = .010). As a sub-analysis, selected characteristics were compared by Aboriginal status to assess for the potential confounding effects of this variable. Respondents who identified as Aboriginal were less likely to have completed high school (p = .013) and to be male (p = .003). Statistically speaking, and at the p<.05 level, Aboriginal and non-Aboriginal respondents did not differ by income source (p = .733), GLBTT status (p = .879), age group (p = .075), HCV prevalence (p = .460), HIV prevalence (p = .243), and whether or not they reported a known IDU in their risk network (p = .238).

### Multivariable Analysis

#### S-IDU and IDU

In model 2 (Table 2) Aboriginal ethnicity (AOR: 2.3, 95%CI:1.8–5.8; p = .017), lifetime syringe sharing after injection (AOR: 2.3, 95%CI: 1.2–3.5) and lifetime T&R use (AOR: 2.6, 95%CI:1.8–5.2; p = .006) were positively associated with S-IDU. The presence of an active IDU in egocentric networks was associated with a threefold higher likelihood of S-IDU group membership (AOR: 3.0, 95%CI:1.6–5.2; p = .003). In model 2 the interaction between female sex and GLBTT status (p = .093) was not significant.

**Table 2:** Unadjusted (UOR) and adjusted odds ratios (AOR) and 95% confidence intervals (95%CI) from multivariable logistic regression models (using generalized estimating equations) examining factors associated with solvent-using injection drug users (S-IDU) vs. injection drug users (IDU) (N = 254).

	Model 1	Model 2
	UOR (95%CI)	AOR (95%CI)
Pathogen Prevalence HCV	2.30 (1.36–3.89)	–
HIV	0.86 (0.41–1.78)	–
		
Age <25	*Ref*	*Ref*
25–29	1.27 (0.47–3.45)	1.91 (0.47–3.45)
30–39	1.89 (0.85–4.19)	2.39 (0.85–4.19)
40+	1.48 (0.72–3.07)	2.79 (0.72–3.07)
		
Female	1.40 (0.82–2.37)	0.91 (0.82–2.37)
		
GLBTT	1.22 (0.62–2.40)	2.24 (0.62–2.40)
		
Aboriginal	3.25 (1.82–5.78)	2.26 (1.82–5.78)
		
Has an IDU in network who has used injection drugs in last 6 months	2.96 (1.64–5.33)	2.97 (1.64–5.33)
		
Shared syringe with someone after injection (lifetime)	2.04 (1.18–3.52)	2.26 (1.18–3.52)
		
Injected Talwin & Ritalin (lifetime)	3.04 (1.79–5.17)	2.63 (1.79–5.17)
		
Injected morphine (lifetime)	0.55 (0.32–0.93)	0.52 (0.32–0.93)

IDU: Injection drug users; GLBTT: Gay, lesbian, bisexual, transgendered, and two-spirited.

Model 1: bivariate comparison between variable and S-IDU/IDU; Model 2: multivariable model excluding HIV and HCV status.

## Discussion

In this study of most at-risk populations in Winnipeg, Canada, the highest prevalence for HCV was found among IDU who reported lifetime usage of solvents. Moreover, this study demonstrated that S-IDU were the most likely to name an active IDU as part of their risk network, as well as reporting the highest lifetime prevalence of syringe-sharing.

This study is consistent with a previous 2003 study in our geographic setting, where an increased risk for HCV among S-IDU was detected among Aboriginal IDU [34]. The present study extends the literature by demonstrating that increased risk for HCV is observed irrespective of the Aboriginal status of users, although since 80% of the respondents were of Aboriginal descent, power may have been limited to detect a statistical difference. Unlike the previous study, our present results indicate that HIV infection was negatively associated with S-IDU status. Thus, although HIV and HCV can affect the same populations, our results demonstrate that the specific underlying transmission dynamics of each pathogen results in differences in who is actually infected. Furthermore, the increased likelihood of S-IDU having another active IDU in their network, in combination with more frequent syringe-sharing is potentially an example of micro-level and macro-level factors combining to increase the likelihood of HCV transmission. That is, given the higher prevalence of HCV in IDU generally, and given the higher frequency of syringe-sharing among S-IDU, the “per injection” risk of HCV transmission, when syringes are shared, may be elevated in our group of S-IDU, leading to the higher observed HCV prevalence. Other studies have identified a link between solvent use and risk of STBBIs[42]; possible mechanisms, specific to solvent use include those related to an extremely marginalized and disadvantaged population, such as riskier sexual behavior (e.g., unsafe sexual practices, higher number of sexual partners)[43], and unique risk networks with a high prevalence of pathogens including STBBIs[37]. Studies examining mediating factors explaining the relationship between solvent use and STBBIs are much needed. Among IDU, differential risk for pathogens has been demonstrated widely[13], [21], [44], [45]. Factors such as drug choice, geographic setting and level of vulnerability influence networks, interactions with members of the same or different sub-populations, and routines around drug preparation and equipment usage[13], [44], [46]–[49]. Further work to refine the micro- and macro-level risks of S-IDU, as well as their interactions with known risks is a worthy study endeavour.

Solvent use is associated with individuals from the most socio-economically disadvantaged populations, alongside a disproportionately higher burden of psychiatric and physical morbidities[50]–[57]. The findings from this study align with literature demonstrating a higher burden of infectious diseases among solvent users[42], [58]. Although lifetime use of solvents has been estimated to be as high as 14% among youth in the United States[59], users who progress to habitual use are of particular concern[60]–[62]. Why some progress to chronic use, despite overwhelming social stigma is not known. Given its association with socio-economic deprivation, and the near ubiquitous availability of solvents, it can be surmised that socio-economic vulnerability and marginalization play a large role[43]. An important consequence of this marginalization is the challenge in developing appropriate care interventions, as solvent users can be particularly intransigent to treatment[51], [63].

As the importance of HCV is being recognized, in terms of its contribution to morbidity and mortality[64], and the increasing costs of treatment[65], the prevention of HCV transmission and acquisition is of increasing importance to public health[4], [66], [67]. However, treatment for HCV through the use of pegylated interferon and ribavarin therapy has features that limit its use more broadly, including cost, requiring adherence for up to 48 weeks, and substantial side effects[68], [69]. At the same time that more effective and less toxic antiviral therapies are becoming available, the potential for these treatments to decrease morbidity and premature mortality has been attenuated due to missed opportunities for early diagnosis, barriers to care and poor follow-up[67], [70].

Thus, the heightened vulnerability to HCV shown by S-IDU, the general difficulties in timely diagnosis and treatment of HCV, and the issues inherent in developing interventions appropriate for this marginalized subpopulation combine to present a public health paradox in our locality: those who are most vulnerable for HCV transmission and acquisition are the least likely to be engaged in care, and are also the least likely to commit to (and to qualify for) HCV therapy[69]. Further work to increase access, linkage and retention into care is a priority for this population.

### Marginalized Populations, Maintenance Networks and Epidemic Potential

Advances in STBBI theory have increased our understanding of STBBI epidemics[71]. For instance, observed macro-level STBBI patterns can be thought of as an aggregation of micro-epidemics[28], [72], [73], whereby in any population there exist a variety of networks comprised of individuals with differential potential to intermingle with individuals from other networks[72], [73]. Researchers have categorized these networks into three groups, in order of decreasing prevalence: core transmitters, bridging populations and the general population[28], [74]. Another important concept is that of *epidemic potential* [71], [75]. Here, transmission success can be classified by its potential to remain within certain subpopulations, or to be more widespread. The epidemic potential for a given pathogen in any population can be labeled as *truncated*, *local concentrated* or *generalized*, with truncated epidemics occurring in isolated “high-risk” subpopulations. Mathematical models have shown that in the absence of intensive targeted interventions, STBBIs can be driven into ever harder-to-reach subpopulations that eschew traditional public health services[76]. Thus, pathogens are maintained and circulated amongst members of subpopulations that have low levels of diagnoses and treatment.

With respect to their impact on the health of the public, given their high levels of HCV, the S-IDU group in our study serves as a maintenance network for HCV. Due to marginalization of S-IDU, HCV would likely remain a truncated epidemic. However, given barriers to access and care, HCV prevalence remains high within this subpopulation; thus, any bridging between S-IDU and other risk networks carries a high potential for more widespread transmission, shifting the epidemic potential from a truncated epidemic to one that is local concentrated. Therefore, interventions aimed at marginalized groups like S-IDU serve not only to decrease morbidity and mortality associated with HCV within S-IDU groups, but ultimately can benefit the population at large.

### Strengths and Limitations

Our study had a number of strengths, including the incorporation of HIV and HCV status, social network and behavioural data. We also sought a broad representation of most at-risk populations in Winnipeg, not just focusing on IDU. Thus, comparisons could be made with other high-risk populations in Winnipeg. Our study also had a number of limitations. First, social desirability and recall biases are always an important consideration for self-reported questions. Notwithstanding the research that has demonstrated the accuracy of self-reporting, and the fact that our research team has had long partnerships with organizations working with some of the most at-risk populations involved in the study, these biases cannot be ruled out. Second, relatively few respondents reported recent drug injection or solvent use; thus for the purposes of this study, we decided to use definitions which examined lifetime use. This had an impact on some of the variables we used in our models, such as lifetime syringe-sharing (vs. in the last 6 months). Thus, generalizing these findings to more recent users of either injection drugs or solvents should be made with caution. Finally, the limitations of cross-sectional data should be noted here, including the inability to draw causal relationships between associated variables.

In conclusion, solvent use stands as a proxy for a culmination of unequal life opportunities, sustained inequities, and failure to develop appropriate interventions. Intermixed with injection drug use, S-IDU from our study population are at increased risk of HCV acquisition. Provision of adequate services with respect to screening, diagnosis and treatment of HCV to S-IDU, and other similarly ostracized subpopulations, may result in wider population-level benefits.
